# A pilot study of the effect of ezetimibe for postprandial hyperlipidemia: Erratum

**DOI:** 10.1097/MD.0000000000013967

**Published:** 2018-12-28

In the article, “A pilot study of the effect of ezetimibe for postprandial hyperlipidemia”,^[1]^ which appeared in Volume 97, Issue 46 of *Medicine*, En-Zhong Xue's name appeared incorrectly as E-Zhong Xue. The corresponding author information should be Chun-Li Liu, Department of Cardiology, The People's Hospital of Yan’an, No. 57 Qilipu St, Baota Qu, Yan’an 716000, China (E-mail: chunliliu007@126.com).
